# Depression, psychological distress and Internet use among community-based Australian adolescents: a cross-sectional study

**DOI:** 10.1186/s12889-017-4272-1

**Published:** 2017-04-27

**Authors:** Erin Hoare, Karen Milton, Charlie Foster, Steven Allender

**Affiliations:** 1 School of Health and Social Development, Deakin University, 1 Gheringhap Street, Geelong, 3220 Australia; 2 Global Obesity Centre, Centre for Population Health Research, Deakin University, Victoria, Australia; 3 British Heart Foundation Centre on Population Approaches for Non-Communicable Disease Prevention, Nuffield Department of Population Health, University of Oxford, Oxford, United Kingdom

**Keywords:** Adolescents, depression, psychological distress, Internet use, community-based

## Abstract

**Background:**

There has been rapid increase in time spent using Internet as a platform for entertainment, socialising and information sourcing. This study aimed to evaluate the relationship between duration of time spent using Internet for leisure, depressive symptoms, and psychological distress among Australian adolescents.

**Methods:**

Depressive symptoms were indicated by the youth self-report module from the Diagnostic and Statistical Manual of Mental Disorders Version IV criteria, and psychological distress was measured by Kessler Psychological Distress scale. Internet use was self-reported based on use on an average weekday, and an average weekend day. Multivariate logistic regression models were used to examine the relationship between Internet use and mental health outcomes. Models were adjusted for potential confounders: age; relative level of socio-economic disadvantage, and body mass index.

**Results:**

Adolescents were aged 11–17 years (M = 14.5 years, SD = 2.04 years). Greatest time spent using internet (≥7 h a day) was significantly associated with experiencing depressive symptoms among females (OR = 2.09, 95% CI = 1.16, 3.76, *p* < 0.05), and high/very high levels of psychological distress for male (OR = 2.23, 95% CI = 1.36, 3.65, *p* < 0.01) and female (OR = 2.38, 95% CI = 1.55, 3.67, *p* < 0.01) adolescents.

**Conclusions:**

With current initiatives to improve health behaviours among adolescents to improve physical health outcomes such as overweight or obesity, it is imperative that the reciprocal relationship with mental health is known and included in such public health developments. Internet use may interact with mental health and therefore could be a modifiable risk factor to reach and improve mental health outcomes for this age group. Caution is advised in interpretation of findings, with some inconsistencies emerging from this evidence.

## Background

A recent examination of sedentary behaviour among Australian young people reported that most youth exceeded the daily recommendation of ≤2 h of screen time per day, and stated that limiting screen-based media use among children and adolescents was ‘*virtually impossible’* [1]. Internet use represents one component of the broader category leisure screen – time. It has been suggested that screen-use, typically measured as specifically for leisure (not for school or work-related purposes) may interact with emotional and mental health [2–4]. With a large proportion of adolescents reporting mental health disorders or associated symptomatology [5], and increasing popularity for online activities for entertainment, socialising and information sourcing, quantifying the strength of any possible associations between Internet use and mental health is critical.

The screen-time recommendation of two hours or less, advocated in Australia [6] and abroad [7], has been criticised as unrealistic, due to screens being deeply integrated into modern life. The health impact of using screens has also been questioned, with some researchers suggesting such recommendations being offered despite being counterproductive based on inconsistent research findings to date [8, 9]. In addition, screen use has been suggested to provide opportunity for individuals to experience meaningful entertainment [10]. Inconsistencies in the impact of Internet use specifically was highlighted by the clinical report from the American Academy of Pediatrics [11], and subsequent contradictory research findings [12]. The report identified ‘Facebook Depression’ as a potential outcome of overuse of social media, however subsequent evidence found no direct link between social media use and symptoms of depression. The continued examination of the health correlates of screen-use through epidemiological studies is imperative.

Previous systematic reviews have predominately focused on the relationship between adolescent sedentary behaviour and physical health outcomes such as body composition, physical activity levels and general well-being indicators [13–15]; however mental health outcomes have mostly been overlooked. Our recent systematic review examined evidence for the relationship between sedentary behaviour and mental health problems among adolescents [16]. We identified a range of cross-sectional and prospective studies and our findings showed consistent evidence for the relationship between sedentary behaviour (most commonly operationalized as hours of screen-time for leisure) and both depressive symptomatology and psychological distress, with significant gender specific associations. However, studies were of poor quality, often using derived mental health indicators based on a single item and therefore vulnerable to validity concerns. Sampling methods often lacked randomisation and representativeness, and weaknesses were identified in statistical methods including failing to account for important confounding variables. The evidence to date is significantly limited by such methodological weaknesses.

We identified the need for a comprehensive examination of mental health status of a large, representative cohort of adolescents, and associations with time spent using Internet for leisure. The impact of sedentary behaviours on health is developing as an important area in research for physical health, and it is critical that the mental health related evidence base is at the same time strengthened. Evidence exists for the unique experiences of mental health for male and females and defining such differences is another critical aspect of this research. The associations between problematic Internet use (addiction) and mental disorders among Australian adolescents has recently been reported [17]. However the relationship between duration of time spent online (spanning low to high users) and mental health status requires further investigation.

The aim of this study was to determine the cross-sectional associations between depressive symptoms, psychological distress and time spent using Internet among a community-based, representative Australian adolescent group. This study aimed to answer the research question; what is the relationship between time spent using Internet for leisure and mental health outcomes; depressive symptoms and psychological distress, in a large, representative Australian adolescent group?

## Methods

This study examined data from the Second Australian Child and Adolescent Survey of Mental Health and Wellbeing, conducted during 2013–2014 [5]. The methods summarised in this section have previously been reported [18]. The national household survey aimed to: capture the mental health and well-being of young people across Australia; provide updated national prevalence estimates of common mental disorders; and report current behavioural patterns such as mental health service use and health risk behaviours.

The survey comprised of two parts; face-to-face interviews with the primary carer, and a self-report questionnaire completed by young people aged 11–17 years within participating households. The current study examined data from the adolescents’ self-reported questionnaire responses, and included some demographic information from the carer-reported data.

The national survey involved a random probability-based sample of 5500 young people aged 4–17 years [18]. This sample size was chosen to meet reliability estimates for prevalence of mental disorders and behavioural patterns for males and females aged 4–11 years and 12–17 years. Areas eligible for participation were selected using a multi-stage, area-based sample selection procedure to ensure social and economic proportional representation across Australia. Participation was voluntary and all participants gave informed written consent prior to survey completion.

### Measures

Participating young people completed the questionnaire privately on a tablet computer. Questionnaires comprised various modules, with those relevant to this study reported below. Details on other information contained in the questionnaire have been reported elsewhere [18].

#### Ethics and consent to participate

Ethical approval for this study was granted by Deakin University Human Research Ethics Committee (2016–035). Original ethics for the Second Australian Child and Adolescent Survey of Mental Health and Wellbeing, was provided by the Australian Government Department of Health Ethics Committee and the University of Western Australia Human Research Ethics Committee.

Participation in the survey was voluntary and written consent was required from all participants. Initial verbal consent was obtained from parents or carers for their child’s participation, which was then followed up with paper consent forms from both parents/carers and the young person. Participation was voluntary and all young people were informed that they had the right to withdraw their consent for study participation at any time. Specific protocols were developed to ensure that if any issues arose for participants in response to the survey, there were services available to assist in receiving information and support they may require.

#### Depressive symptoms

Depressive symptoms were measured by the youth self-report module from the Diagnostic and Statistical Manual of Mental Disorders Version IV (DISC-IV) criteria [19]. The DSM-IV criteria specify that: at least five symptoms of depression must be present for a minimum of a two-week period; the symptoms cause clinically significant distress; and the symptoms interfere with the child or adolescent’s normal functioning at school, at home or in social settings. Symptoms of depression measured included low mood, loss of pleasure in daily activities, irritable, significant weight loss or gain, loss of appetite, insomnia or hypersomnia, restlessness, fatigue and loss of energy, feelings of worthlessness and inability to concentrate. The major depressive module of the DSM-IV has shown moderate to good diagnostic reliability, and moderate to very good validity in relation to clinically meaningful symptomatology [19]. Items used to detect depressive disorder showed high internal consistency (Cronbach’s α > 0.70).

#### Psychological distress

Kessler Psychological Distress scale (K10) [20, 21] was used to measure distress based on symptoms of anxiety or depressive symptoms experienced in the previous month. Ten items asking about an individual’s emotional experiences such as ‘*during the last 30 days, about how often did you feel tired out for no good reason?*’, with the following response options; ‘*none of the time’*, ‘*a little of the time’*, ‘*some of the time’*, ‘*most of the time’* or ‘*all of the time’*. Responses were scored 0 to 4, where 0 represented lowest severity (none of the time) and 4 represented highest severity (all of the time). Participants received a score out of 40 with higher scores representing higher levels of psychological distress. Scores of psychological distress were categorised as; 0–5 = low, 6–11 = moderate, 12–19 = high, and 20–40 = very high, as adopted by the Australian Bureau of Statistics based on previous population-based research [22].

The K10 has been shown to be comparative to other mental health instruments such as mental health component of the General Health Questionnaire, Short Form 12 questionnaire, and the Composite International Diagnostic Interview [23]. The K10 has shown moderate reliability in the general Australian population [24]. In this survey data, the K10 Psychological Distress scale showed high internal consistency (Cronbach’s α > 0.70).

#### Internet use for leisure

Internet use indicators were included in the survey in response to developments in technology as a common means for young peoples’ communication, entertainment and for information sourcing. Internet use excluded time spent on school or work related activities. Internet use was described as Internet accessed on a computer, mobile phone or tablet, and including using social media such as Facebook or Twitter, emailing, looking at websites or chatting online. Questions were focused on time using Internet ‘*not related to schoolwork or for work purposes’*. One item asked ‘*on an average weekday approximately how much time do you spend on the internet?*’ and another item asked ‘*On an average day on the weekend approximately how much time do you spend on the internet*?’ Response categories for both items were; less than 1 h, 1–2 h, 3–4 h, 5–6 h, 7–8 h, 9–10 h, 11 h or more. Responses were categorised into 2 h or less, 3 to 6 h, and 7 h or more, for weekday and weekend responses separately. This was based on Australian guidelines for daily screen-time recommendations (two hours or less per day) [6] and the growing prevalence of Internet related disorders that are characterised by a large proportion of the day spent online [25].

In addition, research has indicated both positive and negative mental health impact of screen-use, dependent on time and frequency of use [26]. We therefore recognised the need to uniquely examine the group reporting 7 h or more to gauge differences between low users, those exceeding screen-time based recommendations currently endorsed by the Australian Government, and those considered very high users. We examined Internet use separately for an average weekday and for an average weekend day as done in previous research examining physical activity and sedentary behavioural research among adolescents [27], and for increased potential for future practical applications of findings (e.g, school or home based interventions).

#### Demographics and confounding variables

Participants were asked basic demographics including age and gender as a part of the questionnaire completion. Socio-economic status was taken from the 2011 Australian Bureau of Statistics (ABS) Index of Relative Socio-Economic Disadvantage for the Statistical Area in which the family was living at the time of the survey [28]. The ABS defines socio-economic advantage and disadvantage as access to material and social resources, and their ability to participate in society [29]. The index forms a collection of variables designed to assess level of advantage/disadvantage, including; deprivation, poverty, human capital, and economic, social and political opportunities [29].

Participants were asked to self-report height in metres and weight in kilograms, from which body mass index (BMI) was derived (divided kilograms by height in metres squared). Weight status (thinness/normal weight, overweight/obesity) was calculated using criteria detailed by Cole et al. (2000) [30]. Cole et al. define age and sex specific cut-points that derive from adult BMI equivalent of 25 kg/m^2^ for overweight and 30 kg/m^2^ for obesity. Thinness was combined with normal weight due to the low proportion of adolescents categorised as thin (<6%).

#### Statistical analysis

Analyses were conducted using STATA release V.14.1 (Stata Corp., College Station, Texas, USA, 2015). All variables were checked for missing data. In all cases there were little missing data and case-wise deletion was used where relevant. Descriptive statistics were used to analyse differences between males and females using independent sample Student *t*-tests and Pearson’s χ2 test where appropriate. Separate multivariate logistic regression models were used to examine the relationship between Internet use and dichotomous mental health outcomes; depressive symptoms (symptoms suggesting depression compared to no depression) and psychological distress (high/very high levels of distress compared to low/moderate levels), stratified by gender. Models were adjusted for potential confounders: age; relative level of socio-economic disadvantage (henceforth referred to as socio-economic status [SES]); and body mass index (BMI). Results were considered statistically significant at *p* < 0.05.

## Results

The survey ran during May 2013 and April 2014. Participant characteristics are reported in Table 1. A total of 2967 adolescents completed the self-report questionnaire and were subsequently included in this study. Adolescents were aged 11–17 years (M = 14.5 years, SD = 2.04 years) and females formed approximately half the sample (48.4%). Overall mean BMI was 21.2 (SD = 4.5) and approximately a quarter (25.9%) of participating adolescents were classified as overweight/obese. Other demographic characteristics indicated that this sample was broadly representative of the wider Australian population when compared to data from the Census of Population and Housing [18].

**Table 1:** Characteristics of survey sample

	Number	Percent
Gender		
Male	1530	51.6
Female	1437	48.4
Age		
11–15 years	1615	54.4
16–17 years	1352	45.6
Mean age in years (SD)	14.6	(2.0)
Index of relative socio-economic disadvantage		
Lowest quintile (least advantaged)	473	15.9
Second quintile	554	18.7
Third quintile	543	18.3
Fourth quintile	676	22.8
Highest quintile (most advantaged)	721	24.3
Parent or carer education		
Bachelor degree of higher	911	32.4
Diploma or certificate	1268	45.2
Secondary education	631	22.4
Weight status		
Normal	2030	74.1
Overweight/obesity	711	25.9
BMI Mean SD		
Mean (SD)	21.2	(4.5)

Self-reported mental health findings indicated that 9.7% of youths experienced depressive symptoms, with a significantly greater proportion of females (13.9%) compared to males (5.8%) classified with symptoms indicating depression (*p* < 0.05) (Table 2). Gender differences were found in average depressive symptomatology scores with females reporting greater total number of symptoms (M = 4.5, SD = 5.9) compared to males (M = 2.8, SD = 4.4). One fifth (21.7%) of adolescents experienced high/very high levels of psychological distress, however a greater proportion of females (28.7%) reported high/very high levels of psychological distress compared to males (15.3%).

**Table 2:** Proportion of male and female adolescents with major depressive disorder, depressive symptomatology and psychological distress, and time spent using Internet

	Total	Males	Females	*p* ^1^
Major depressive disorder^a^				
With depression *n* (%)	288 (9.7)	89 (5.8)	199 (13.9)	*p* < 0.05
Total depressive symptomatology score Mean (SD)	3.6 (5.3)	2.8 (4.4)	4.5 (5.9)	*p* < 0.05
Psychological distress^b^				
High/Very high	645 (21.7)	233 (15.3)	412 (28.7)	*p* < 0.05
Total psychological distress score Mean (SD)	7.6 (6.8)	6.4 (5.8)	8.9 (7.6)	*p* < 0.05
Internet				
Uses Internet n (%) Yes	2941 (99.2)	1516 (99.2)	1425 (99.2)	*NS*
Internet use on an average weekday n (%)				*NS*
2 h or less	1248 (42.4)	670 (44.2)	578 (40.6)	
3–6 h	1170 (39.8)	576 (38.0)	594 (41.7)	
7 h or more	523 (17.8)	270 (17.8)	253 (17.8)	
Internet use on an average weekend day n (%)				*NS*
2 h or less	1015 (34.5)	547 (36.1)	468 (32.8)	
3–6 h	1253 (42.6)	628 (41.4)	625 (43.9)	
7 h or more	673 (22.9)	341 (22.5)	332 (23.3)	

*NS* Non-significant

^1^Tested for significant difference between males and females in mental health measure using Chi goodness of fit test or Independent samples *t*-test as appropriate

^a^Major Depressive Disorder measured by the youth self-report module from the Diagnostic and Statistical Manual of Mental Disorders Version IV (DISC-IV) criteria

^b^Psychological distress measured by the Kessler Psychological Distress scale

Almost all adolescents (99.2%) reported using the Internet. Over half (57.6%) of the adolescent group reported using the Internet three or more hours on an average weekday, and 65.5% reported three or more hours of Internet use an average weekend day. Close to one fifth reported 7 or more hours of Internet use on an average weekday (17.8%) and on an average weekend day (22.9%). No significant differences existed between Internet usage patterns between male and female subgroups.

Unadjusted logistic regression models demonstrated a significant relationship between experiencing depressive symptoms and higher reported hours of Internet use among females (Table 3). Compared to those reporting two hours or less, female adolescents reporting 3–6 h of Internet use on a weekday were twice as likely to experience depressive symptoms (OR = 2.11, 95% CI = 1.35–3.29, *p* < 0.05). Females reporting seven hours or more on a weekday (OR = 2.33, 95% CI = 1.35–4.02, *p* < 0.05) or weekend (OR = 2.25, 95% CI = 1.32–3.83, *p* < 0.05) had greater increased odds of depressive symptoms. Female adolescents who used the Internet for seven or more hours on an average weekday were more likely to report high/very high levels of psychological distress (OR = 1.73, 95% CI = 1.15–2.60, *p* < 0.05), as were both males and females reporting more than two hours Internet use on an average weekend day.

**Table 3:** Unadjusted (Model 1) and adjusted (Model 2) associations for Internet use for major depressive disorder, depressive symptomatology, and psychological distress (adjusted for age, SES and BMI)

	Model 1						Model 2^a^					
	Males			Females			Males			Females		
	*OR*	*95% CI*	*P*	*OR*	*95% CI*	*p*	*OR*	*95% CI*	*p*	*OR*	*95% CI*	*P*
Major depressive disorder^b^												
Weekday internet use												
≤ 2 h	Ref			Ref			Ref			Ref		
3–6 h	1.27	0.70, 2.33	0.429	**2.11**	**1.35, 3.29**	**0.001**	1.20	0.63, 2.26	0.581	**1.87**	**1.15, 3.02**	**0.011**
≥ 7 h	1.99	0.97, 4.04	0.057	**2.33**	**1.35, 4.02**	**0.002**	1.81	0.86, 3.83	0.120	**2.09**	**1.16, 3.76**	**0.014**
Weekend internet use												
≤ 2 h	Ref			Ref			Ref			Ref		
3–6 h	0.95	0.51, 1.73	0.861	1.33	0.83, 2.10	0.232	0.94	0.50, 1.78	0.847	0.93	0.57, 1.51	0.779
≥ 7 h	1.62	0.80, 3.27	0.177	**2.25**	**1.32, 3.83**	**0.003**	1.47	0.70, 3.07	0.306	1.62	0.93, 2.82	0.088
Age							**1.20**	**1.04, 1.37**	**0.010**	**1.38**	**1.24, 1.53**	**0.000**
SES^c^							1.02	0.87, 1.19	0.795	0.96	0.86, 1.08	0.521
BMI							**1.06**	**1.00, 1.11**	**0.020**	1.02	0.98, 1.06	0.269
Psychological distress^d^												
Weekday internet use												
≤ 2 h	Ref			Ref			Ref			Ref		
3–6 h	1.00	0.69, 1.46	0.986	1.36	0.99, 1.86	0.055	0.93	0.62, 1.39	0.725	1.16	0.83, 1.62	0.390
≥ 7 h	1.53	0.97, 2.43	0.068	**1.73**	**1.15, 2.60**	**0.008**	1.45	0.89, 2.37	0.135	1.54	1.00, 2.39	0.051
Weekend internet use												
≤ 2 h	Ref			Ref			Ref			Ref		
3–6 h	**1.47**	**1.00, 2.16**	**0.050**	**1.54**	**1.11, 2.13**	**0.009**	1.39	0.93, 2.08	0.437	1.39	0.98, 1.97	0.065
≥ 7 h	**2.28**	**1.43, 3.68**	**0.001**	**2.67**	**1.78, 4.01**	**0.000**	**2.23**	**1.36, 3.65**	**0.002**	**2.38**	**1.55, 3.67**	**0.000**
Age							1.03	0.95, 1.12	0.437	**1.20**	**1.11, 1.29**	**0.000**
SES							1.06	0.95, 1.17	0.302	0.98	0.89, 1.07	0.603
BMI							**1.03**	**1.00, 1.07**	**0.047**	**1.04**	**1.01, 1.07**	**0.008**

Bold indicates significant values (*p* < 0.05)

OR Odds Ratio BMI Body mass index SES Socio-economic status

^a^Adjusted for age, relative level of socio-economic disadvantage and BMI

^b^Co-efficient = Odds Ratio for depression compared to no depressive disorder

^c^Socio-economic disadvantage indicated by 2011 Australian Bureau of Statistics Index of Relative Socio-Economic Disadvantage for the Statistical Area in which the family was living at the time of the survey, where higher index level indicates greater level of advantage

^d^Co-efficient = Odds Ratio for high or very high psychological distress compared to low/moderate psychological distress

After adjusting for potential confounders (age, SES, and BMI), the relationship between Internet use and depressive symptoms remained significant for female adolescents reporting 3–6 h (OR = 1.87, 95% CI = 1.15–3.02, *p* < 0.05) and seven or more hours (OR = 2.09, 95% CI = 1.16–3.76, *p* < 0.05) on an average weekday. Psychological distress appeared more frequent among female adolescents reporting seven or more hours Internet use on a weekday, however this relationship did not reach significance (*p* = 0.051). Seven or more hours of Internet use on the weekend doubled the odds of experiencing high/very high psychological distress for males (OR = 2.23, 95% CI = 1.36–3.65, *p* < 0.05) and females (OR = 2.38, 95% CI = 1.55–3.67, *p* < 0.05) compared to adolescents reporting two hours or less.

Age increased likelihood of depressive symptoms in both males (OR = 1.20, 95% CI = 1.04–1.37, *p* < 0.05) and females (OR = 1.38, 95% CI = 1.24–1.53, *p* < 0.05), and psychological distress in females (OR = 1.20, 95% CI = 1.11–1.29, *p* < 0.05). BMI was positively associated with depressive symptoms among males (OR = 1.06, 95% CI = 1.00–1.11, *p* < 0.05) and psychological distress in both males (OR = 1.03, 95% CI = 1.00–1.07, *p* < 0.05) and females (OR = 1.04, 95% CI = 1.01–1.07, *p* < 0.05).

## Discussion

### Principal findings

The aim of this study was to examine depressive symptoms and psychological distress in a large, representative cohort of Australian adolescents, and to identify associations with time spent using the Internet for leisure purposes. This study found that females (13.9%) were more likely to experience depressive symptoms compared to males (5.8%), and this is consistent with previous estimates [31]. High or very high levels of psychological distress were experienced by a greater proportion of females (28.7%) compared to males (15.3%) and this gender difference is also consistent with previous estimates [32].

Most adolescents reported using the Internet for three or more hours on an average weekday (57.6%) and on an average weekend day (65.5%). A recent examination of Australian adolescents [33] reported that most (84.6%) exceeded Australian Government guidelines or two hours or less daily hours spent using screens for leisure, and this current finding was therefore not overly surprising. Nevertheless, this finding demonstrates the proportion of adolescents adhering to the current Australian guidelines of two hours of less is low, given the adolescents participating in this study exceed this recommendation based on Internet use alone.

Compared to adolescents reporting Internet use for two hours or less, depressive symptoms were most frequent among higher users of the Internet (3 or more hours per day) among females only. This finding is novel in light of previous literature which has reported higher levels of depression among high internet users in combined male and female adolescent populations [3, 34–36]. This finding is particularly important given significantly higher rates of depression among adolescent females compared to males, and the need for novel treatment and prevention initiatives. Previous research has found associations between body image disturbances and self-objectification with time spent using social media sites among female adolescents [37]. Given the cross-sectional design of the current study, causality cannot be speculated. However, it is possible that negative self-esteem may increase with time using such sites and precede depression. It is also possible that depressed individuals may seek comfort through connection and social support through such sites.

Using the Internet for seven or more hours on an average weekend day doubled the odds of reporting high or very high psychological distress among males and females, compared to those using the internet for two hours or less. Psychological distress is distinguished from depressive symptoms as it includes symptoms of both depression and anxiety. The current study found this relationship significant only among the group reporting the highest level of Internet use (seven or more hours) on an average weekend day, compared to those reporting two hours or less. This finding is novel in light of previous literature which found associations between psychological distress and internet use among community-based adolescents, when internet use exceed two hours a day threshold [38, 39]. This finding may reflect increasingly normalisation of internet use for day-to-day activities, and may also reflect increased opportunity for online mental health support as previously described.

Mental health concerns are known to increase from younger to older adolescents [40], and this was represented in the current findings with age predicting increased levels of depressive symptoms in both males and females and psychological distress in females. Increased BMI was linked to depressive symptoms among males, and psychological distress in both males and females. Previous research has supported the relationship between increased BMI, overweight/obese weight status, and mental health among young people [33, 41–44].

Asides from the described findings, there were no other significant associations between Internet use and mental health among this sample of Australian adolescents. While the significant findings support the ongoing investigation into the relationship between Internet use and mental health, such relationships were inconsistent. The possibility for positive or null relationship between mental health and Internet use, as described in the background section, remains plausible.

#### Potential mechanisms

Given the cross-sectional design of this study it is not possible to provide conclusion on causality and therefore mechanism cannot be approximated. The following discussion of potential mechanisms are provided only as possible explanations, and may support future longitudinal research enquiry.

While female adolescents are known to experience increased risk for internalising disorders including depression and anxiety [45], the finding that 13.9% were suffering depressive symptoms, and close to a third (28.7%) experienced high or very high levels of psychological distress, is cause for great public health concern. It has been reported that during adolescence, females are at greater risk of factors associated with mood and anxiety-related disorders [45]. Females may experience consequential negative feelings towards the self in the development of such disorders, which then exacerbates symptoms and disorder trajectory [45]. Sociological perspectives examine gender roles and practices as contributors to disparities in health, such as social expectations of masculinity and femininity, and suggest that such practices have the potential to be modified to improve mental health outcomes [46]. The finding that increased Internet use was associated with depressive symptoms requires further exploration; however, given that adolescent females experience significant increased risk for depressive symptoms overall, this could be an important intervention point for future research.

While cross-sectional design precluded conclusions on causality, symptoms of depression such as low motivation and worthlessness, may prohibit adolescents engaging in healthy lifestyle behaviours. Physical activity data were not collected and may have been a confounding variable. Being physically active assists with building self-efficacy, resilience and offers social integration, and it has been suggested that such characteristics may protect against depression [47–49]. Importantly, the relationship between physical activity and sedentary behaviour is complex. The findings of a recent systematic review concluded that the association between sedentary behaviour and physical activity in children and young people is negative, but small, suggesting these behaviours do not directly displace one another [50]. The findings reported in this study would have benefitted in controlling for physical activity levels, to determine whether there is unique contribution of Internet use to mental health among adolescents. Furthermore, young people engage in many sedentary behaviours that are widely approved (e.g., reading for leisure, homework/studying) and it is unrealistic to expect all activities considered sedentary to be associated to negative health outcomes.

In addition, sedentary behaviour is characterised as activities involving very low energy expenditure, and pathophysiological processes occurring during time spent using the Internet may have a negative impact upon mood and emotional health outcomes [51]. Our previous systematic review reported some evidence for the relationship between screen-time and negative mental health outcomes, independent of physical activity [38, 52], therefore this mechanism needs further investigation. An additional consideration is that the relationship between types of screen use is uncertain in that young people may displace television viewing as they watch programs online, and may or may not include this as ‘time spent online’. Furthermore, the Internet has been identified as a useful information source for young people experiencing mental health problems and time spent online may be attributable to help-seeking behaviour [53]. While our findings indicated some significant relationships, we advise caution in applying such findings to sedentary behaviour overall, and we recommend that screen use is investigated by type and content of activity, as opposed to an overall composite screen time measure.

Although the relationship between BMI, weight status and mental health has been previously reported [54], this has historically been most commonly found among female adolescent populations [55, 56]. The findings of this study suggest that this relationship is more pronounced among male adolescents, and this builds on recent trends showing similar vulnerabilities for overweight and obese males [33, 43]. Traditionally, support for weight-based and body image concerns have been directed towards females [57]. It is possible that male adolescents experiencing overweight or obesity experience negative mental health outcomes, however may not receive the same mental health support. Additionally, overweight and obese males may be less likely to recognise symptoms for depression, and seek mental health support [45].

Finally it is possible that adolescents experiencing depressive symptoms or increased levels of psychological distress may turn to online sources for support, leading to increased time spent online. The opportunity for internet based mental health interventions for young people has previously been supported, with results suggesting significant positive effect for computer-delivered cognitive behavioural therapy for anxiety and depression symptoms [58]. Given high levels of habitual Internet use among Australian young people, initiatives that are delivered online may hold unique opportunity to improve health outcomes by utilising existing common behaviours.

#### Strengths and limitations

This study was strengthened by a large, community-based Australian adolescent sample that was representative of the Australian population on key demographic characteristics. The measures used for depressive symptoms and psychological distress have both previously been shown to demonstrate good reliability and validity among adolescent populations. Analyses was strengthened by controlling for potential confounders; age, relative level of socio-economic disadvantage, and BMI, which have been suggested to impact on internet use behaviours and mental health among adolescents [59–62]. Despite inclusion of these covariates, this study was limited by the lack of available physical activity data, and the potential confounding impact on the relationship between internet use and mental health. Furthermore, current health status was not included in the data and failure to include this as a covariate may have confounded the results.

This study was limited by cross-sectional design that precluded conclusions on the directional relationship between duration of time spent using Internet, depressive symptoms and psychological distress. Time estimates for Internet usage were derived from single questions on typical self-reported behaviour, and are therefore subject to various methodological weaknesses such as recall bias, or social desirability bias. Furthermore, the internet use item stipulated that estimates were for time spent using internet other than work or school purposes. It is possible that associations between internet use for non-leisure purposes and mental health factors may exist, but were not captured in this study. This study was also limited by the use of self-report measures; however objective measures have been shown to pose feasibility issues for large, community based samples. While our analyses were conducted following stringent statistical methodology, it has been suggested that in some instances odds ratios may be fragile to Type I error and should be interpreted with caution [63].

#### Directions for future research

These results suggest that identifying mechanisms driving gender specific risks for mental health is of utmost importance. Time spent using Internet could be included as an indicator for potential mental health concerns, particularly among adolescents spending large proportions of time online on an average day. With the known benefits of healthy lifestyle behaviours for physical and emotional health, further research could examine the impact of reducing time spent online on mental health disorders and associated symptoms – although online activity for young people is ubiquitous and decreasing usage may be unrealistic [1]. An important step in this research is, therefore, to examine the type and duration of online activity, and the behaviours that time spent online replaces, to further identify mechanisms at work in this relationship. Positive mental health outcomes of Internet use need to be further explored, due to important inconsistencies (e.g, non-significant relationships) found in this research, and potential to leverage on common existing lifestyle behaviours.

## Conclusion

There is a concerning number of adolescents with depressive symptoms and a large proportion of this current adolescent sample reported experiencing high or very high levels of psychological distress. Generating evidence to inform efforts to prevent and treat mental health disorders is a clear public health priority for this age group. Most adolescents in this cohort exceeded Australian guidelines for daily screen-time recommendations of two hours or less per day, and this was through Internet use alone (recommendations includes other screen-time such as watching television or gaming). With current developments in initiatives to improve health behaviours among adolescents to improve physical health outcomes such as overweight or obesity, it is imperative that the relationships with mental health are known and included. Internet use may interact with mental health and therefore could be a modifiable risk factor to reach and improve mental health outcomes for this age group. Some inconsistencies emerged from this study, and further investigation is needed to determine the prospective relationship between Internet use and mental health, and the possibility of positive or null relationships.
